# The General Practitioner and the Mental Health Problem

**Published:** 1918-12

**Authors:** George A. Hastings

**Affiliations:** Executive Secretary, Committee on Mental Hygiene, New York State Charities Aid Association


### ORIGINAL ARTICLES.

### "The General Practitioner and the Mental Health Problem."

By George A. Hastings,

EXECUTIVE SECRETARY, COMMITTEE ON MENTAL HYGIENE, NEW YORK STATE CHARITIES AID ASSOCIATION.

It might be said that when a layman undertakes to write for a medical journal he has fosaken his own field and trespassed upon another to which only his temerity could have invited him. But four years' service as the executive of a committee whose medical personnel has exercised brilliant leadership in an organized effort for the better understanding and control of mental disorders has made it seem that general practitioners might conceivably be interested in the subject of the mental health of the community from the standpoint of someone outside the profession.

Medical men everywhere have noted the increasing importance which the problem of mental disease and defect has assumed in the popular and scientific mind within the past few years. To be sure, the organized movement for improved methods of control and treatment of mental disorders developed much later than the public health movement in the realm of physical diseases. Yet the past decade has seen remarkable progress in bringing the public and the medical practitioner to see the need of a better knowledge of the mental health problem, and wiser and more efficacious measures of dealing with it.

With 225,000 committed insane in this country, and with at least as many feebleminded, a very large proportion of whom are still unprotected in the community, the gravity of the problem of mental disorder is all too obvious. If anything were needed to supplement our experience along this line in civil life, it is furnished by the disclosures of the prevalence of mental diseases and defect through the operation of the military draft. The United States, for the first time in the history of nations, selecting its military forces on a basis of mental as well as physical fitness, has rejected approximately 15,000 men as unfit because of nervous and mental disorders, and of this number about 5,000, or roughly one-third, were feebleminded.

### THE ORGANIZED ATTACK ON MENTAL DISORDERS.

The last ten or a dozen years have seen the inauguration of National, State and community efforts, to obtain a better understanding of the problem of mental disorders, to promote individual and community mental hygiene, and to obtain more scientific and adequate facilities for treatment, prevention and after care. Thus far, the mental specialists—the psychiatrists and neurologists—have stood the brunt of the fight against mental disorders so far as the medical profession is concerned. Here again, as in so many other directions, preventive medicine has come to the aid of the community in a manner which has already wrought inestimable public benefit and reflected credit on the skill and public spirit of the profession. National, State and local committees for mental hygiene, with laymen in their membership, but with policies formulated and directed by medical specialists, have made exceedingly substantial progress toward acquainting the public with the essential facts regarding mental diseases and defects and in stimulating a deeper sense of responsibility for better control of these menaces to the health and efficiency of the population.

Yet the very fact that the specialists in the fields of neurology and psychiatry have assumed so much of the responsibility on behalf of the profession has had a tendency to prevent the average general practitioner from assuming his rightful role and responsibility in dealing with the problem. One could scarcely blame the men in general practice thus far for leaving much of the work in this highly specialized field to the men who have made a special study of it and who have demonstrated distinguished qualities of skill and leadership in it. Yet my experience in helping to carry out the mental health program under medical direction in New York State, has convinced me that the next stage of progress toward the solution of the problem will depend in a very large degree upon the conception which general practitioners gain of the movement, and the amount of effort they lend in carrying the mental health gospel and the modern facilities of prevention and treatment to the very doors of the people. The general practitioner can do most efficient service in this direction because he retains to a much greater extent than the specialist that personal relation between doctor and patient upon which so much of the usefulness of the profession depends.

IN THE FIELD OF MENTAL DISEASES.

Perhaps too much has sometimes been claimed as possible in the way of prevention of mental disorders. Yet there is the authority of the most conservative and distinguished of mental specialists for the statement that a very substantial proportion of mental disorders are preventable in some stage of their development. Without being too deeply concerned whether the percentage of preventable mental disorders is 25 or 40 per cent, there is abundant evidence to sustain a conclusion that the field of prevention is exceedingly large and promising and that the inculcation of personal and community mental hygiene and unremitting emphasis on the value and imperativeness of early diagnosis and treatment will accomplish some of the results for which we are striving.

And who can do more toward establishing and carrying out these fundamental concepts in everyday life than the general practitioner? He it is who comes in contact with the family and the intimate family life from the beginning to the end of life's span. He it is who has the confidence of the family and on whose judgment and counsels, as well as skill, the family puts its reliance. A word from the competent general practitioner whose alertness has detected the beginnings of mental disorder has more weight than volumes of printed matter and scores of mental hygiene papers and exhibits. If he realizes the significance of the symptoms, if familiar with the facilities for early diagnosis and treatment, and can successfully interpret to the family the patient's need of frank and early attention to a mental disorder as he would in a case of physical disease, he will have become a most useful and practical exponent of mental hygiene. If, enlarging his sphere from the patient and the family to the community, he can help interpret the new social psychiatry in terms of every-day need, and the facilities to meet that need, his usefulness is increased a thousandfold.

Is every general practitioner familiar with the hospitals existing in his State, his county or his community where mental disorders are treated? Does he know whether these institutions have outpatient department clinics, where persons may go at stated times and receive competent advice, diagnosis, and treatment from specialists while they remain in their own homes? Does he know whether the schools are giving sufficient attention to the problem of the mental as well as the physical health of the child? Are they helping the child early in life to make satisfactory adjustments and adaptations to his daily surroundings and problems and aiding him to develop his personality and to overcome tendencies toward seclusiveness, peevishness, or viciousness, which may be but symptoms of something far worse, and but the beginnings of a long series of failures to adjust which will inevitably spell complete failure later in life?

Does the general practitioner show the interest which he should, as a doctor and a citizen, in seeing that his own community provides the right kind of care and treatment while patients with mental disorders are undergoing observation or waiting transfer to an institution for mental disorders? Will the physician permit his community to treat a sick person—mentally sick—by locking the patient in the local jail, or the pest house, while he is waiting removal to the State or other hospital for mental diseases? He would scarcely think of thus treating a pneumonia case in its critical stages!

What more valuable service can the general practitioner do, especially in the larger community, than to help bring about the establishment of a local psychiatric hospital where the mild and short-term cases of mental disorder can be cared for in their own community instead of being removed to distant and usually large hospitals? Such a center is not only a place of first aid treatment and safe temporary care, but it becomes a center of prevention, study and research into the causes and treatment of mental disorders.

Whose voice will have more weight than that of the "family doctor" when it is lifted up in the effort to bring about a more wholesome public attitude toward mental diseases? We are gradually discarding the terms "lunatic" and "lunatic asylum," and mental diseases are losing much of the stigma which these unfortunate terms have helped to keep alive. A proper public attitude toward the mental patient and toward the institution for his treatment have a very important bearing upon the standards of care and treatment which prevail.

And take the cases of beginning paresis and incipient dementia praecox and many other forms of mental disorder. How much misgiving, misery, and damage might often be avoided if the general practitioner had a wider knowledge of these disorders and was always on the alert to detect the symptoms and to take prompt and appropriate steps?

One, of course, hesitates to suggest more subjects in the curriculum of the medical college, even as he is reluctant to suggest adding more duties to the busy general practitioner. Yet it is impossible to escape the very earnest conviction that the average medical college today devotes far too little attention to mental disorders in preparing men for general practice, and that the average general practitioner has scarcely begun to realize his usefulness and his responsibility in matters of mental health. In a very large number of cases, as Dr. Thomas W. Salmon has pointed out, patients owe far more to the skill and insight of the general practitioner who recognizes danger when it first threatens than to the specialist who applies the means of cure.

"The most important lessons," Dr. Salmon says, "is that the general practitioner should be constantly on the watch for mental diseases in the homes which he visits, in the consulting room, the dispensary, and the hospital ward. If he is watchful he will be certain to observe many such cases, and if he observes early cases of mental diseases, he will have an opportunity of performing a most valuable service by securing for them the timely treatment which is so esssential. * * * Psychiatric clinics, psycopathic wards in general hospitals, and simpler means of commitment for observation or treatment, all will bring mental cases to the psychiatrist earlier, but in a vast number of cases the issue is decided earlier yet."

If the general practitioner will thus serve the community he will become the greatest single ally of the mental hygiene movement. No organized movement for mental health, whether conducted by State, National, or local organizations, can attain its greatest usefulness without him.

### IN THE FIELD OF MENTAL DEFECT.

The problem of feeblemindedness, too, is serious—as serious perhaps as the insanity question. The enormous prevalence of mental defectiveness and the failure of the community thus far to deal with it in an adequate and organized way are finally bringing the public to a realization of the gravity of the situation and the urgency of more effective means to control and mitigate the menace of uncared for defectives.

Community control of the feebleminded involves the progressive steps of identification, registration, instruction, supervision, and segregation. As in the case of mental disease, the problem of mental defect is to a considerable extent a medical and scientific problem. Without the aid of the specialist in formulating programs, and of the general practitioner in translating these programs into results, we can scarcely hope for any great degree of progress.

But I am convinced that with the profession and the public aroused to grapple with the question, and with the increasing knowledge on which to base programs we have reasons to believe that we may be at the threshold of a new era in dealing with the feebleminded.

To be sure there is not yet complete agreement as to the program. Many questions of policy and procedure remain to be settled, but it can safely be said that we have gotten away from the idea that all of the feebleminded should be shut up in institutions of the traditional type. Colony care merits further study and experiment. The importance of instruction and supervision of defectives in special classes and in their own homes is increasingly recognized. Then too, without means of identification and a system of registration, we can know neither whom to segregate or supervise or instruct. Reliable diagnosis and the establishment and patient carrying out of a system of registration until we know the whereabouts, the kind of defect, and the family and personal history of the registrants, are necessary to progress toward dealing effectively with them.

Whatever the individual may feel regarding the potency of sterilization in diminishing feeblemindedness, certainly it is a fact that public opinion generally has not yet advanced to a point where it will sustain any widespread application of this remedy. But, on the other hand, there is a general agreement that, with perhaps eighty per cent of mental defect due to heredity, we will never strike at the deepest roots of the problem until parenthood of the unfit is restricted. As Dr. Walter E. Fernald has said, "Parenthood is not for all."

Eugenics—real eugenics—has an important part to play in the prevention and control of mental defect. There is need of a public conscience regarding the marriage of the unfit, regarding the protection of the feebleminded in the community, who, left to themselves and their own weak volitions, are the easy prey of the unscrupulous, a source of moral contagion, physical diseases. crime, and an unending stream of their kind to burden succeeding generations.

It is almost superflous to mention in detail what the general practitioner can do to help deal more wisely and effectively with the feebleminded. In his every day practice, in his touch with the profession, his interest in the schools, the courts, and the social agencies of his community, he has an opportunity to help immeasurably in getting sound programs of relief adopted and put into effect. There is no organization or individual working for a solution of the problem who does not greatly desire the interest and active help of the general practitioner.

---

Some people overlook a small account when they would not allow a note to remain unpaid a day after it is due. A large part of the revenue for a medical publication comes from small subscription accounts. The publisher of the Texas Medical Journal needs every dollar that is due her, and needs it now. Suppose you, unless you have already paid, sit right down and mail your check for a year or two year's subscription.

